# A Systematic Review of Machine Learning Techniques in Hematopoietic Stem Cell Transplantation (HSCT)

**DOI:** 10.3390/s20216100

**Published:** 2020-10-27

**Authors:** Vibhuti Gupta, Thomas M. Braun, Mosharaf Chowdhury, Muneesh Tewari, Sung Won Choi

**Affiliations:** 1 Michigan Medicine, Department of Pediatrics, University of Michigan, Ann Arbor, MI 48109, USA; 2 School of Public Health, Department of Biostatistics, University of Michigan, Ann Arbor, MI 48109, USA; tombraun@umich.edu; 3 Michigan Engineering, Computer Science and Engineering, University of Michigan, Ann Arbor, MI 48109, USA; mosharaf@umich.edu; 4 Michigan Medicine, Department of Internal Medicine, Hematology/Oncology Division, University of Michigan, Ann Arbor, MI 48109, USA; mtewari@med.umich.edu; 5 Center for Computational Medicine and Bioinformatics, University of Michigan, Ann Arbor, MI 48109, USA; 6 Michigan Engineering, Department of Biomedical Engineering, University of Michigan, Ann Arbor, MI 48109, USA

**Keywords:** machine learning, artificial intelligence, sensors, mobile health, mHealth, hematopoietic stem cell transplantation, HSCT

## Abstract

Machine learning techniques are widely used nowadays in the healthcare domain for the diagnosis, prognosis, and treatment of diseases. These techniques have applications in the field of hematopoietic cell transplantation (HCT), which is a potentially curative therapy for hematological malignancies. Herein, a systematic review of the application of machine learning (ML) techniques in the HCT setting was conducted. We examined the type of data streams included, specific ML techniques used, and type of clinical outcomes measured. A systematic review of English articles using PubMed, Scopus, Web of Science, and IEEE Xplore databases was performed. Search terms included “hematopoietic cell transplantation (HCT),” “autologous HCT,” “allogeneic HCT,” “machine learning,” and “artificial intelligence.” Only full-text studies reported between January 2015 and July 2020 were included. Data were extracted by two authors using predefined data fields. Following PRISMA guidelines, a total of 242 studies were identified, of which 27 studies met the inclusion criteria. These studies were sub-categorized into three broad topics and the type of ML techniques used included ensemble learning (63%), regression (44%), Bayesian learning (30%), and support vector machine (30%). The majority of studies examined models to predict HCT outcomes (e.g., survival, relapse, graft-versus-host disease). Clinical and genetic data were the most commonly used predictors in the modeling process. Overall, this review provided a systematic review of ML techniques applied in the context of HCT. The evidence is not sufficiently robust to determine the optimal ML technique to use in the HCT setting and/or what minimal data variables are required.

## 1. Introduction

### 1.1. Background: Machine Learning (ML)

Machine learning is an application of artificial intelligence (AI) that provides machines the capability to automatically learn and improve from experience without being explicitly programmed [1,2]. It is a natural extension of traditional statistical approaches [3], focusing primarily on predictions, automatically identifying patterns within data, and performing tasks beyond human capabilities (i.e., classification of images) [4]. Applying machine learning (ML) algorithms on given data include building a model (i.e., learning relationship between the data features and outcome), then validating (i.e., tuning the model parameters) and testing the model (i.e., applying the tuned model on a new testing dataset to make predictions and evaluations). The predictions improve with more experience (i.e., training data). Some ML applications include autonomous vehicles [5], automated detection of diabetic retinopathy on retina images [6], identification of skin lesions on skin images [7], Google translate [8], and Facebook facial recognition [9]. Overall, the most important component for applying ML algorithms is data, which must be abundant to build robust and generalized predictive models.

An enormous amount of healthcare data is being generated nowadays from electronic health record (EHR) systems that include medical diagnosis, prescriptions, lab test results, imaging data, vital signs, and patient demographics. [10,11]. Multi-omics (e.g., genomics, proteomics) data further enhance complexity. However, data analytics of these multidimensional data using ML techniques potentially open avenues for early diagnosis, prognosis, as well as treatment of diseases [3]. Moreover, such novel data analytics may inform clinical decision-making sooner and more efficiently in efforts to prevent life-threating diseases.

### 1.2. Applying ML Techniques in the Context of Hematopoietic Stem Cell Transplantation (HSCT)

Hematopoietic stem cell transplantation (HSCT) [12] is a potent form of immunotherapy that is used for treatment of various malignant and non-malignant hematological malignancies (e.g., leukemia, lymphoma, and multiple myeloma). Despite recent advances, HSCT remains a complex procedure that includes high-dose chemotherapy followed by infusion of autologous stem cells or from a related or unrelated human leukocyte antigen-matched (HLA-matched) donor (e.g., allogeneic) [13]. Hematopoietic stem cell transplantation recipients face numerous post-transplant related complications, including life-threatening infections, graft-versus-host-disease (GVHD), and relapse or recurrence of disease, which may lead to morbidity and mortality [14].

Machine learning techniques have the capability to extract patterns from large amounts of complex data and build predictive models, to automate various tasks in the HSCT context, such as selecting the appropriate donor for a HSCT recipient, identifying biomarkers for early diagnosis of post-HSCT complications, and GVHD risk stratification modeling. However, there are challenges: First, HSCT is a complex procedure that involves numerous post-transplant complications such that selecting the appropriate ML technique is not straightforward. Second, the real-time data capture of various post-HSCT complications as well as other clinical, demographic, and/or genomic data variables for algorithm training and predictive modeling can be limited. Third, the captured data are typically dispersed among various data stores (e.g., EHR, cloud storage, individually managed databases), which require integration and standardization before ML algorithms can be applied. Lastly, the evolution of disease states during HSCT further complicate the process of applying ML techniques as predictive models may become obsolete at given time points.

There are several advantages of using ML techniques in HSCT, such as rapid and early diagnosis of various post-HSCT diseases (e.g., GVHD), efficient donor selection, personalized patient care, and reduced costs-of-care.

The motivation behind this manuscript was to better understand the existing use of ML applications as well as provide research directions for potentially developing new data analytics techniques in the field of HSCT. Thus, herein, we sought to systematically review the published literature of ML techniques currently being applied in the HSCT setting. We specifically examined the type of data streams included, specific ML techniques used, relevant predictors identified, and type of clinical outcomes measured. Moreover, we discuss the current challenges in applying these existing ML techniques in HSCT settings and provide future research directions to possibly tackle such challenges. The goal of this review is not to analyze ML techniques applied for all hematological malignancies, but to specifically focus on recent work in pre-and post-HSCT settings, which has not yet been previously reported in the literature, according to our knowledge. In doing so, we then conclude the review with a current case study of an mHealth app (Roadmap 2.0) that incorporates wearable sensors to highlight the current challenges in the field as well as propose potential novel ML techniques that could be applied in analyzing the data output. Different supervised, unsupervised, and reinforcement learning techniques used in the reviewed studies are shown in Supplementary Figure S1 and described in Supplementary Table S1.

This review is organized according to the following sections: Section 2 (Methods) describes existing methods used in the systematic review, and Section 3 (Results) presents the systematic review findings. Section 4 (Discussion) discusses the reviewed methods, including current challenges and future research directions. Section 5 (Case Study) describes a case study of a proposed mHealth platform that addresses existing challenges and offers possible robust solutions. Finally, Section 6 (Conclusions) concludes the paper.

## 2. Methods

### 2.1. Search Strategy

**Figure 1:**
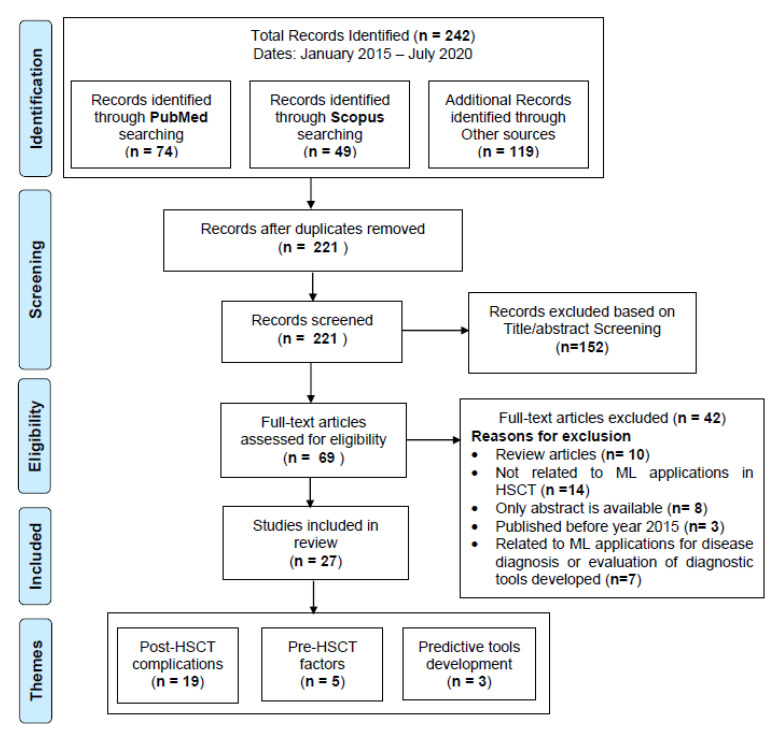
PRISMA Workflow for systematic identification of scientific literature.

We conducted a systematic review of articles written in English, using the following online literature databases: PubMed, Scopus, Web of Science, and IEEE Xplore, following the Preferred Reporting Items for Systematic Reviews and Meta-analysis (PRISMA) guidelines [15]. The workflow diagram for the systematic identification of scientific literature is shown in Figure 1 Search terms included different combinations of keywords related to “HSCT” and “machine learning” joined by Boolean operators “OR” (to combine the terms related to specific domain) and “AND” (to combine the terms from different domains). A search query using the terms listed in Table 1 was used for the retrieval of primary studies: The search was performed on July 2020 using the above search query. It was targeted to retrieve recent articles from the last five years (January 2015–July 2020), identifying current state-of-the-art ML techniques being applied in HSCT.

### 2.2. Study Selection

We started our search with the search query (Table 1) on the databases listed above. The title and abstracts of the resulting studies were first screened to identify the studies related to ML applications in HSCT settings. After identifying the eligible studies, additional inclusion-exclusion criteria were applied to retrieve the primary studies of our review (details are provided in Figure 1).

Studies were eligible if they fulfilled the following inclusion criteria in our review: (1) focused on ML techniques in HSCT (i.e., specifically on pre-and post-complications of HSCT); (2) written and published in English; (3) published between January 2015 and July 2020; (4) full text available rather than abstracts; (5) original studies published in peer-reviewed journals or appeared in conference proceedings.

Studies were not eligible if they fulfilled the following exclusion criteria in our review: (1) review articles rather than primary research; (2) conducted system analysis without incorporating ML techniques for predicting post-HSCT complications; (3) used ML algorithms to diagnose specific hematological malignancies; (4) used comparison and evaluation of prognostic score tools for risk stratification models. The identified studies meeting the inclusion criteria were subsequently categorized into three broad categories based on major themes identified: (1) post-HSCT complications, (2) pre-HSCT factors, and (3) predictive tools development.

### 2.3. Data Extraction and Evaluation

The data were extracted from all studies meeting our inclusion criteria for the review. It consists of tables containing study information (e.g., authors’ name, title, year of study); type of data streams included (e.g., clinical, genomic, physiological, other longitudinal data streams); sample size (e.g., number of participants); type of ML techniques used (e.g., ensemble, decision tree, SVM, regression techniques); and clinical outcomes measured (e.g., risk prediction, survival, relapse) (Table 2). The data for all studies were extracted independently by 2 authors (VG and SWC) by mutual agreement and discrepancies were resolved by discussion with other authors (TB, MC, and MT). The extracted data were finally evaluated by all authors independently.

## 3. Results

We identified 242 articles in the identification phase. Twenty-one duplicate articles were removed to produce 221 articles for title and abstract screening. We further excluded 152 articles in the title and abstract screening and accessed full text of remaining 69 articles for further evaluations in the eligibility phase. Finally, 27 articles met our inclusion criteria and were considered as primary studies for this review, as shown in Figure 1. Out of 27 primary studies, 18 were from PubMed, three from Scopus, and six from other databases.

**Figure 2:**
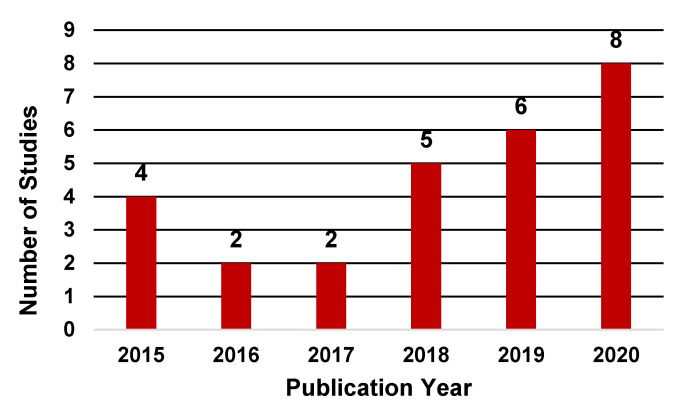
Distribution of studies by publication year.

In terms of publication years, the application of ML techniques in the HSCT setting has steadily increased in recent years. As shown in Figure 2, the number of studies published between 2018 and 2020 examining the application of ML techniques in HSCT contributed to 70% of the total studies with only eight studies published before 2018. Notably, the number of studies published through July 2020 was greater than the total studies published during years 2016 and 2017.

We have broadly categorized the ML techniques into nine categories. Supplementary Figure S1 shows the taxonomy of ML techniques used in the reviewed studies. Each broad category contains a specific subcategory of ML technique. The majority of studies fell under four broad ML categories: ensemble learning (63%), regression (44%), Bayesian learning (30%), and support vector machines (SVM) (30%). More details are provided in Supplementary Table S2 and Figure S2.

Clinical data (e.g., stem cell source, conditioning regimen, graft type, blood characteristics) was the most frequently used data stream in the studies. Eighty-five percent of studies used clinical data and the remaining 15% used other types of data streams. As shown in Table 2, out of 27 studies, 15 used only clinical data, while the rest used clinical with genomic and biological data (eight), imaging data (two), and other types of data (e.g., demographics, gene expression) (two). In terms of data sources, registries were used in nine of the studies, five studies used other databases and EHR as data source, six used the hospital data, while a data source was not found/clear in seven of the studies.

### 3.1. Major Themes Identified

Three major themes were identified: (1) post-HSCT complications; (2) pre-HSCT factors; and (3) predictive tools development. These were further subcategorized based on clinical outcomes. Post-HSCT complications comprised studies related to modeling the risk of survival/death, relapse, and GVHD. Pre-HSCT factors and predictive tools development included tasks, such as the identification of appropriate donors for HSCT, detection of pre-HSCT infections, and development of risk prediction tools to facilitate personalized clinical care. Not surprisingly, more than half of the studies (19) were categorized into post-HSCT complications, followed by pre-HSCT factors (five) and predictive tools development (three).

#### 3.1.1. Post-HSCT Complications

Studies belonging to risk of death and relapse post-HSCT (12) were predominant compared with studies related to risk stratification (two) and disease diagnosis (six). Research on death and relapse prediction studies utilized different clinical, genomic, demographics and transplant features to first build a predictive model from a training set and then apply to a validation/test set to predict the likelihood of death and relapse in a pre-specified time duration (e.g., six months, 12 months, two years, five years) post-HSCT.

Lu et al. [16] presented a deep learning approach (attention based bi-directional long short-term memory [Att-BLSTM]), utilizing clinical and genomic features, in order to predict the likelihood of relapse and survival/death in patients with acute myeloid leukemia (AML). For time- dependent clinical and genomic features, they collected blood test results (e.g., complete blood count (CBC), white blood count (WBC)), information about medications, and time of HSCT with relapse and gene mutation. For static features, they considered demographics (e.g., age, gender) and cytogenetics test at diagnosis. A total of 65 features were used for predictions. Best performance was achieved, while predicting likelihood of death and relapse for the next 0–3 months. Another study [17] analyzed data of 217 patients with acute leukemia to predict relapse within one year of HSCT using ADT. However, major clinical and demographic factors considered here for building predictive model were age, diagnosis (e.g., AML), rDRI (refined disease risk index) [43], donor type, graft source, use of total body irradiation, and conditioning regimen. Goswami et al. [18] applied a stacked ML technique to predict relapse within 36 months of autologous HSCT in patients with multiple myeloma (MM). They applied spectral clustering [44] to cluster patients into low- and high-risk groups (i.e., relapse within 36 months as high-risk otherwise low-risk) and then fast and frugal trees [45] to further calibrate relapse risk groups.

There are some studies [19,20] that applied ML techniques to identify specific genes responsible for relapse in patients undergoing HSCT. Ritari et al. [19] performed a genomic-wide-sequencing of active immunoregulatory regions, whole exome, and MHC regions on 151 HSCT recipients with HLA matched donors. They employed random forest classification model to identify the genetic variants associated with the relapse risk. The results showed that germline genetic variations were highly associated with relapse. Marino et al. [20] identified 19 amino acid substitutions associated with risk of adverse outcomes following HSCT using ML techniques. The outcomes included incidence of grade III-IV acute GVHD, disease-free survival, and overall survival. Random forest and logistic regression models were constructed using donor-recipient clinical characteristics (e.g., recipient age, gender match, type of disease, disease stage) and all candidate amino acid substitutions. None of the identified substitutions were associated with high-risk outcomes when validated on an independent cohort.

Recently, Arab Yarmohammadi et al. [21] proposed an algorithm to detect post-HSCT relapse in AML patients using automated image analysis. Bone marrow Wright–Giemsa aspirate slides were collected from 39 AML patients and a deep learning algorithm was employed to segment myeloblasts (i.e., a cell type in bone marrow to characterize AML). Shape and texture features were subsequently extracted from the segmented blasts and feature selection algorithm were applied to generate top features for the predictive model. Three predictive models were developed using random forest and linear discriminant analysis to predict the likelihood of relapse across patients.

Some prior works [22,23] utilized reinforcement learning to improve the prevention and treatment of acute and chronic GVHD. Krakow et al. [22] also used reinforcement learning to develop an adaptive treatment strategy (ATS) for immunosuppressive management while treating acute GVHD that would maximize disease-free survival two years post-HSCT. Liu et al. [23] used deep reinforcement learning to accomplish three tasks: (1) treatment after transplant; (2) prevent GVHD; and (3) treatment of acute and chronic GVHD. It comprised of a deep neural network for predicting the expert actions and a deep reinforcement learning to choose actions and update Q-value estimates. The results provided promising accuracy in predicting human expert decision and implementing reinforcement learning.

Studies have also investigated the use of ML applications in prediction of death post-HSCT. Shouval et al. [24] proposed an in-silico (i.e., iterative computerized simulations) AI based approach for predicting non-relapse mortality (NRM) within 100 days post-HSCT and improving the prediction accuracy affected by the quality of features, statistical methods, and population size, etc. This study was a large-scale multi-center study with a total of 26,266 patients. Various ML algorithms were applied, such as AdaBoost, Logistic Regression (LR), Random Forest (RF), Alternating decision tree (ADT), and Naïve Bayes (NB). The stage of disease, type of donor, and conditioning regimen were the major clinical variables identified for predicting the outcome. In another study [25], ADT was used for predicting death within 100 days post-HSCT and focused on the prediction of leukemia-free survival, NRM, and overall survival at two years.

Predicting the risk of post-HSCT complications (e.g., GVHD) using ML techniques was investigated in some studies [26,27]. Tang et al. [26] examined 324 allogenic HSCT recipient data from the EHR using penalized logistic regression to predict risk of grade II–IV acute GVHD. They utilized longitudinal vital sign features (e.g., body temperature, heart rate, respiratory rate, diastolic and systolic blood pressure, peripheral capillary oxygen saturation) along with patient demographics and donor and transplant characteristics for building the predictive model. The investigators found that temperature, systolic blood pressure and features representing longitudinal trends were the most significant features for predicting risk of acute GVHD. Another study [27] developed a predictive model for predicting acute GVHD (grades II–IV and III–IV), using ADT. Pre-transplant donor and recipient clinical characteristics were used for the prediction model. Interestingly, using mouse model systems, Kuang et al. [28] developed an early diagnostic model of acute GVHD. They analyzed continuous temperature profiles using principal component analysis and k-means clustering and captured temperature differences post-HSCT between mice that developed acute GVHD and those that did not. Their results suggested that continuous body temperature may signal early acute GVHD.

Lopez et al. [29] used random forest to identify genes associated with the incidence of chronic GVHD while Sharifi et al. [30] used unsupervised methods to distinguish pulmonary complications post-HSCT. Gandelman et al. [31] also utilized unsupervised machine learning techniques for risk stratification of chronic GVHD. They first converted multidimensional transplant recipient data into two dimensions using vi stochastic neighbor embedding (viSNE), then applied a self-organizing maps (SOM) algorithm for patient clustering based on organ scores. Risk scores were the computed for the resulting clusters and overall survival was calculated for the identified risk groups of patients with chronic GVHD. Sharafeldin et al. [32] identified genetic biomarkers to predict cognitive impairment post-HSCT using elastic net regression. The authors built and compared three risk prediction models using different set of variables (i.e., sociodemographic, clinical, and combined, including genetic variables). Significant associations were identified between gene variants and cognitive impairment post-HSCT. Gene signature was investigated by Cocho et al. [33] using ML techniques to diagnose GVHD-dry eye. Shrinkage discriminant analysis, SVM and k-nearest neighbor algorithms were used for gene signature identification that identified four genes with significant predictability of GVHD-dry eye.

Leclerc et al. [34] proposed an ML approach to predict appropriate cyclosporine drug dosage after pediatric HSCT to prevent acute GVHD. The authors employed a Bayesian learning model on clinical and biological data collected from 155 pediatric patients. Their model identified the best dosing regimen reaching therapeutic range after HSCT.

#### 3.1.2. Pre-transplant Factors

Selecting an appropriate donor-recipient pair is challenging in HCT field. Overall, 30% of patients in United states find HLA-matched sibling donors [46], while the remaining rely on public registries to identify unrelated donors. An essential condition for an unrelated donor to be genetically compatible for the recipient, is to match at least eight out of 10 of the recipient’s HLA alleles along with other donor characteristics. Moreover, determining the availability of unrelated donors is also a key concern. Therefore, research has been done to automate the donor selection and availability tasks using ML techniques [35,36,37,38]. A recent study by Li et al. [35] proposed an ML approach to predict donors’ availability by considering five years of donor information as input variables and responses to verification typing (VT) requests as outcome variables. VT requests consist of a set of tests to verify the donor’s identity and concordance. The authors found that the boosted decision tree technique performed well in predicting donor availability with an Area Under curve (AUC) of 0.826 compared with other techniques (e.g., LR, SVM). In another study [36], the authors proposed an ML approach to predict the availability of registered donors and found that donor availability could be determined by using demographics and non-genetic factors.

For donor selection algorithms, some studies [37,38] utilized SVM for building a predictive model to identify appropriate unrelated donors. Buturovic et al. [37] developed a model to prioritize donors as preferred or not preferred based on the five-year survival status of their recipient, while Sivasankaran et al. [38] developed a model to select optimal HLA-matched donors based on donor characteristics and historical choice behavior. However, the authors in [37] were unsuccessful in predicting the unrelated donor. Finally, Brasier et al. [39] utilized ML techniques for diagnosis of pre-HSCT infections (i.e., Aspergillus) in immunocompromised patients undergoing chemotherapy. They utilized the ensemble algorithm generalized path seeker for identifying molecular biomarkers that correlated with infection.

#### 3.1.3. Predictive Tools Development

Few studies [40,41,42] were identified regarding AI based tools to facilitate decision-making and personalized treatment. Lee et al. [40] developed a risk prediction tool using super learner technique to predict risk of grade III-IV acute GVHD. They considered two binary outcomes: (1) diagnosis of grade III-IV acute-GVHD within 100 days post-HSCT; and (2) composite outcome considering both whether the patient developed acute-GVHD or died within 100 days post-HSCT. The tool was validated in 9651 patients who underwent unrelated donor HSCT. Okamura et al. [41] developed a web application tool for personalized prognosis prediction post-HSCT using an ensemble algorithm (random survival forest). Their tool provided functionalities to plot the prognosis prediction curves for one-year overall survival, progression-free survival, relapse, and NRM, by using patient-specific pre-transplant factors that can be adjusted according to the patients’ characteristics. Leclerc et al. [42] developed a decision support tool to predict optimal cyclosporine drug dosing, which is a major immunosuppressant for preventing GVHD. Authors utilized Bayesian networks for building predictive models.

## 4. Discussion

Herein, we systematically reviewed ML techniques currently being applied in the HSCT setting. The findings suggest a remarkable increase in its application over recent years. This is likely due to multiple factors, including advances in technology, such as better computing and storage capabilities (e.g., cloud computing), broad-scale implementation of EHRs, adoption of smart technologies (e.g., wearable sensor technologies, mobile health platforms (mHealth)), and emergence of more powerful and automated ML techniques (e.g., deep learning, stream learning). Additionally, the results indicate that the majority of ML applications were related to post-HSCT complications, such as risk prediction of survival/death and relapse in patients undergoing HSCT or of post-HSCT complications (e.g., acute/chronic GVHD, infections) (Table 2). Clinical and genetic data were the most commonly used predictors in the primary studies since these data were largely associated with clinical HSCT outcomes.

SVM, RF, BN, ADT, and LR were the most commonly used ML techniques in the published HSCT studies. SVM was used for its robustness to noise and high dimensional data. SVM performed the best in two studies where it was used for selecting an appropriate donor for the HSCT recipient [36,37]. Donor selection is challenging due to highly imbalanced data, which can be handled by cost sensitive SVM, as in [38]. However, one of the major drawbacks of SVM is its “black-box” nature due to its generated model that cannot be interpreted easily by clinicians, possibly reducing the clinical applicability of SVM.

Our review found that the RF technique was the only technique used in two of the studies for identifying significant genes associated with HSCT outcome [19,29]. Random forest can potentially reduce overfitting and generate an accurate model. This technique can also rank features based on relevance to the outcome. Thus, enabling one to rank highly significant genes associated with the HSCT outcome. However, drawbacks of RF include complexity, including interpretability of the findings. Interestingly, we found that BN was used in two studies where they were used to predict optimal cyclosporine drug dosing to prevent GVHD [34,42]. The unique ability of BN to encode domain knowledge and consider various variability factors make them attractive in the HSCT setting. However, the requirement of discretizing input variables could be a major drawback. ADT technique was used in four of the reviewed studies where it was used primarily to predict survival/death and relapse post-HSCT [17,24,25,27]. ADT outperformed RF model in one study [16,20,24,26,35,36,40]. Finally, logistic regression was used in six of the primary studies as a comparator technique, but it did not outperform any of the other ML techniques.

Some of the other best performing ML techniques used in the primary studies were BDT, GBM, Att-BLSTM, SL, and generalized path seeker (GPS). All of these models belong to ensemble category, which combines multiple models to improve performance, reduces overfitting, and eases the modeling of high-dimensional datasets. However, longer training times and non-interpretability limit these models in clinical settings. Overall, evidence is not sufficiently robust to determine the optimal ML technique to use in the HSCT setting. Importantly, due to dynamic characteristics unique to each study, it remains challenging to identify an optimal ML technique that can be applied robustly across all conditions. However, our results suggest that ADT technique could be useful in the field of HSCT due to their interpretability which is crucial in the clinical settings as shown in primary studies [17,24,25,27]. The findings were generalizable, robust, and clinically relevant.

Our review suggests that despite the increase in ML techniques applied to HSCT studies in recent years, challenges remain with major gaps in the literature that need to be addressed. In doing so, there is exciting opportunity to develop and apply novel analytics in the field. Table 3 highlights some of these challenges, which may potentially lead to future research directions that address these challenges and Table 4 discusses the summary of limitations identified in the reviewed studies with the potential solutions to overcome those limitations.

Importantly, our systematic review identified a gap in application of ML techniques in mHealth platforms along with wearable sensors technology. To our knowledge, using the search terms listed in Table 1, there were no published studies that examined multi-parameter data streams (e.g., clinical, genomic, psychosocial, physiological) using new or emerging ML techniques. Thus, based on the compiled evidence-based literature, we present a case study for a proposed mHealth platform (Roadmap 2.0) that could address some of the major challenges discussed above, which hints toward a potential robust solution. Limitations of our review process may have included restriction to original research published in English language as well as the reporting period (2015–2020).

## 5. Case Study: Roadmap 2.0

“Roadmap 2.0” is an mHealth platform designed for real-time capture of large volumes of wearable sensor data (e.g., heart rate, sleep, activity/steps) through a Fitbit API (https://dev.fitbit.com/build/reference/web-api/), longitudinal health outcome measures (e.g., survey questionnaires [patient-reported outcomes, PROs], EHR data variables), and interaction with a multi-component app, including positive psychology-based exercises (https://roadmap.study/). This study is registered on ClinicalTrials.gov (NCT040984844). In the proposed clinical trial [50], large, multi-parameter data streams will be generated, utilizing clinical, physiological, psychosocial, demographics, EHR datasets. For example, clinical data will consist of patient biological variables (e.g., age, gender, race/ethnicity), demographics (e.g., marital status, education, employment), health variables (e.g., comorbidities), disease variables (e.g., disease, disease risk); PRO data will collect health-related quality of life (HRQOL) information from questionnaires; sensor data will collect physiological information (e.g., sleep, activity/steps, heart rate). These data streams will be captured and stored in secure HIPAA-compliant servers where initial exploratory analyses will be performed, including descriptive statistical summary of clinical data variables, scoring of PROs according to manuals, classical biostatical techniques that assess patient outcomes (e.g., Kaplan–Meier survival [51] outcomes, Fine and Grey [52] competing risks of GVHD/relapse), and searching motifs in physiological data streams. Qualitative data of HSCT semi-structured interviews will also be generated in efforts to identify themes or patterns of patient views and perspectives.

Data analytics could include signal processing and supervised ML techniques. A single high dimensional feature vector will be generated by combining the PRO scoring, statistical summaries, and Motif search features in the feature extraction part of the data analytics pipeline. These features will act as an input to potential ML techniques applied. These ML techniques could be considered depending on the following cases: (1) perform analytics on the collected data at a later time point; (2) continuously perform real-time analytics on the collected data and learn from the data; (3) perform analytics efficiently to save resources and processing time. For each of the mentioned cases, the type of ML technique as well as its application will be different. In the first case, four ML techniques could be considered: (1) penalized LR; (2) linear SVM; (3) boosted trees; and/or (4) RF due to their large applicability in HSCT settings, as shown in this review. Additionally, these techniques are suitable in batch scenarios where predictive models are built on the assumption of static data distribution and model update frequency is longer. Moreover, there are no limits on processing time to produce results. However, in the second case, since data will require continuous processing (i.e., real-time or near real-time), results will need to be generated faster. In this case, the ML technique should process data in real-time, track dynamic changes in data distribution and update the model accordingly. Thus, online/incremental learning algorithm, such as NB, Hoeffding trees [53], LR with stochastic gradient descent [54] and OzaBoost [55] could be used. For the third case, online learning algorithms and distributed stream analytics platforms, such as Apache Spark [56], Storm [57], and Flink [58] could be useful since large amount of data can be processed in minimal time and memory. Moreover, distributed platforms may be useful as the number of individuals using mHealth platforms, like Roadmap 2.0, will likely continue to rise.

**Figure 3:**
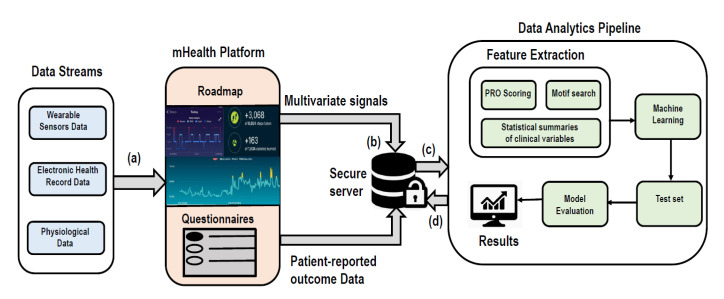
Schematic workflow of Roadmap 2.0. Firstly, (**a**) large volumes of wearable sensor (i.e., Fitbit) data stream (e.g., heart rate, sleep, activity/steps), Electronic health Records and physiological data streams will be captured in real-time in mHealth platform Roadmap 2.0. The captured multi-parameter data streams (**b**) will be stored in secure HIPPA compliant server. It will contain multivariate physiological signals and patient reported outcomes data (generated from patients’ response of survey questionnaires). (**c**) The stored data will be processed in data analytics pipeline. Here, firstly features will be extracted from all diverse types of data and then machine learning algorithms will be used to build a predictive model. This model will be applied to test set for predictions on the unseen data. Finally, the predictive model will be evaluated using AUC. Also, feature importance will be computed. (**d**) The final results will be stored in the secure server.

A general analytics workflow (Figure 3) with the mHealth platform, Roadmap 2.0, could begin with the division of the dataset into training and testing sets (80/20) with cross validation for parameter-tuning. An appropriate ML technique will be applied on the training set based on the above three cases, and the trained model can then be applied to the test set for classification of patients with unseen labels. Finally, the model will be evaluated by computing area under curve (AUC). Moreover, feature importance will be calculated to identify features most responsible for the given outcome. This framework may address some of the major challenges identified in this review. Firstly, it provides a continuous data capture using wearable sensors (e.g., Fitbit), which alleviates the limited data capture issue. Secondly, it provides a secure comprehensive data store (i.e., secure HIPPA compliant repositories) of longitudinal, multi-parameter data that may facilitate real-time data access. Thirdly, identifying patterns in high-dimensional, multivariate time-series may further enable capture and real-time analyses of large clinical data. A supervised learning algorithm will be used, similar to the approaches described in the primary studies of this review.

## 6. Conclusions

We conducted a systematic review of ML techniques in the HSCT setting. In recent years, there has been increasing interest in the application of ML techniques in healthcare. The majority of studies used supervised ML techniques (e.g., ensemble learning), related to post-HSCT complications, but were limited by small numbers of patients. None of the studies provided robust evidence to determine an optimal ML technique for HSCT or minimal number of variables required to build predictive models. However, our results suggest that ADT could be applicable in HSCT setting due to their interpretability. Our findings suggest possible improvement by selecting a larger sample size, evaluation measures, and modelling validation sets. Our review did not identify any published studies incorporating ML applications for wearable sensors or mHealth platforms. The described Roadmap 2.0 case study showed there may be potential for such technologies to address certain limitations in healthcare through longitudinal monitoring of patients. This review provides a current-state-of-the-art application of ML techniques in the healthcare domain, particularly HSCT, discusses current challenges, provides potential future research directions, and presents a case study to address challenges through technology.

## Figures and Tables

**Table 1:** Search query for the retrieval of studies.

(HSCT OR HCT OR GVHD OR acute GVHD OR aGVHD OR leukemia OR lymphoma OR autologous HCT OR allogeneic HCT OR Hematopoietic Cell Transplantation OR Bone marrow transplant OR Hematopoietic cell transplant OR Hematopoietic stem cell transplantation OR Graft-versus-host disease) AND (Machine Learning OR Artificial Intelligence).

**Table 2:** A brief summary of reviewed studies.

Reference	No. of Participants	Data Streams Used	Outcomes	Best ML Technique	Compared ML Techniques	Major Theme Identified
Lu et al., 2019 [16]	637	Clinical, genomic & demographics	AML 2-years survival and relapse, mortality	Att-BLSTM	SVM, LR	Post-HSCT complications
Fuse et al., 2019 [17]	217	Clinical	Risk of Leukemia relapse after 1 year of allo-HSCT	-	ADT	Post-HSCT complications
Goswami et al., 2019 [18]	347	Clinical	Relapse risk within 36 months of autologous-HSCT	-	Stacked ML	Post-HSCT complications
Ritari et al., 2018 [19]	161	Clinical & genomic	Genomic biomarkers for relapse risk of various hematological malignancies for allo-HSCT recipient	-	RF	Post-HSCT complications
Marino et al., 2016 [20]	2107	Clinical	High-risk amino acid substitutions and position types for grade III-IV acute-GVHD, TRM, disease free survival	-	RF, LR	Post-HSCT complications
ArabYarmohammadi et al., 2020 [21]	39	Images	Relapse risk in AML patients post-HSCT	-	Deep learning, LDA	Post-HSCT complications
Krakow et al., 2017 [22]	9563	Clinical	Adaptive treatment strategies	-	RL	Post-HSCT complications
Liu et al., 2017 [23]	6021	Clinical	Optimal Dynamic treatment regimes	-	Deep RL	Post-HSCT complications
Shouval et al., 2016 [24]	26,266	Clinical	NRM 100 days post HCT in acute leukemia	-	NB, ADT, LR, MLP, RF, AdaBoost	Post-HSCT complications
Shouval et al., 2015 [25]	28,236	Clinical	Overall Mortality 100 days post-HSCT	-	ADT	Post-HSCT complications
Tang et al., 2020 [26]	324	Clinical	Grade II-IV acute-GVHD risk	-	L2 regularized LR	Post-HSCT complications
Arai et al., 2019 [27]	26,695	Clinical	grade II-IV & III-IV aGVHD risk	ADT	NB, MLP, RF, Ada- boost	Post-HSCT complications
Kuang et al., 2019 [28]	28	Clinical & sensor	Non-invasive biomarkers for acute-GVHD diagnosis in mice	-	PCA, k-means	Post-HSCT complications
Serrano-López et al., 2020 [29]	29	Genomic	Gene biomarkers for chronic-GVHD diagnosis	-	RF	Post-HSCT complications
Sharifi et al., 2020 [30]	66	Images	Differentiate among pulmonary complications post-HSCT	-	k-means + SVM	Post-HSCT complications
Gandelman et al., 2019 [31]	339	Clinical	Classify patients with chronic-GVHD according to organ scores	-	k-means	Post-HSCT complications
Sharafeldin et al., 2020 [32]	277	Clinical, genomic & demographics	post-BMT cognitive impairment	-	ENR	Post-HSCT complications
Cocho et al., 2015 [33]	36	Clinical & genomic	Genomic biomarkers for GVHD associated Dry eye	SVM	k-NN, SDA	Post-HSCT complications
Leclerc et al., 2018 [34]	155	Clinical & biological	initial cyclosporine dose blood concentrations Post-HSCT	BN	NB, SVM, RF	Others
Li et al., 2020 [35]	10,258	Clinical & Demographics	Donor availability	BDT	LR, SVM	Pre-HSCT factors
Sivasankaran et al., 2018 [36]	Not clear	Demographics & member related factors	Donor availability	GBM	SVM, LR	Pre-HSCT factors
Buturovic et al., 2018 [37]	1255	Clinical	Selecting appropriate unrelated donor for patients undergoing HSCT	-	SVM	Pre-HSCT factors
Sivasankaran et al., 2015 [38]	3035	Clinical	Selecting appropriate unrelated donor for patients undergoing HSCT	SVM	k-NN, CART	Pre-HSCT factors
Brasier et al., 2015 [39]	68	Clinical	Detection of pre-HSCT infection in patients undergoing chemotherapy	GPS	RF, CART, MARS	Post-HSCT complications
Lee et al., 2018 [40]	9651	Clinical	Grade II-IV agvhd risk or death within 100 days post-HSCT	SL	LR, BRT, MARS, BART, RR, ENR, ANN	Predictive Tools Development
Okamura, et al. 2020 [41]	363	Clinical	1-year overall survival, PFS, relapse, and NRM	-	RSF	Predictive Tools Development
Leclerc et al., 2020 [42]	211	Clinical & biological	Best first cyclosporine dose	-	BN	Predictive Tools Development

**Abbreviated Terms:** BDT: Boosted Decision Tree; LR: Logistic Regression; SVM: Support Vector Machine; HSCT: Hematopoietic stem cell transplantation; GVHD: Graft-versus-host-disease; RF: Random Forest; AML: Acute Myeloid Leukemia; Att-BLSTM: Attention Bidirectional Long-short-term-memory; PCA: Principal Component Analysis; NRM: Non-relapse mortality; NB: Naïve Bayes; MLP: Multilayer Perceptron; AdaBoost: Adaptive Boosting; GPS: Generalized Path Seeker; CART: Classification and Regression Tree; MARS: Multivariate Adaptive Regression Spline; ADT: Alternating Decision Tree; ENR: Elastic Net Regression; BN: Bayesian Network; SL: Super Learner; GBM: Gradient Boosting Machine; BRT: Boosted Regression Trees; BART: Bayesian Additive Regression Tree; RR: Ridge Regression; ANN: Artificial Neural Network; SDA: Shrinkage Discriminant Analysis; LDA: Linear Discriminant Analysis; RSF: Random Survival Forest; SM: Stacked Model; PLR: Penalized Logistic Regression; k-NN: k-nearest Neighbor.

**Table 3:** Summary of challenges in applying ML techniques in HSCT.

Challenges	Reasons	Potential Solution
Limited Data Capture	■ Complex HSCT procedure with numerous post-transplant complications ■ Lack of continuous and real-time capture of various data streams involved ■ Mix of automated and manual data capture	■ Utilize wearable sensor devices or leverage mHealth platforms for robust data collection
Data Quality Issues	■ Lot of missingness and inconsistencies due to complex data collection procedures ■ Loss of important variables lead to loss of relevant information	■ Developing autonomous, adaptive, and online preprocessing algorithms that can automatically capture the data quality issues and resolve them by employing appropriate techniques in real-time
High Dimensional Data	■ Large number of clinical and/or genomic variables associated with the HSCT outcome	■ Developing novel streaming dimension reduction techniques for efficient processing of large number of features associated with the HSCT outcome
Data Privacy Issues	■ Large amount of sensitive patient data is required in building predictive models due to numerous factors involved ■ Combining multiple data streams from disperse data stores leads to potential data privacy issues	■ Developing appropriate privacy measures, such as data anonymization techniques to ensure complete privacy of patients’ data ■ Using technique such as “federated learning” [47] that trains a shared global model via a centralized aggregation server, while keeping sensitive data in local institutions of their origin ■ Enabling some form of privacy access control to different data streams that can ensure that only those with proper authorization can access a patient’s data streams
Obsolete Predictive Models	■ Dynamic evolution of disease states in patients undergoing HSCT	■ Developing adaptive ML techniques having capability of detecting data changes over time and adapting accordingly
Diverse Data Types	■ Captured data are of different modalities and sampled at different rates	■ Multi-modal data integration techniques using deep learning has to be developed for effective integration
Data Integration issues	■ Most of the captured data are typically dispersed among various data stores (e.g., cloud storage, EHR, individually-managed databases)	■ Using mHealth platforms could be a potential solution.

**Table 4:** Summary of limitations of reviewed studies.

Limitations	Consequences	Potential Solution
Lack of interpretable predictive models	■ Biased results ■ Lack of generalizable models ■ Non-applicability to clinical decision making	■ Development or application of more interpretable ML techniques such as ADT in HSCT setting ■ Utilizing methods such as shapely additive explanations (SHAP) [48] and Local Interpretable Model-Agnostic Explanations (LIME) [49] for better interpretability ■ Better data visualization techniques
Lack of model validation	■ Leads to non-generalizable models	■ Use of validation sets to check initial errors of the built model and calibrate the model further before applying it to test sets
Smaller sample size	■ Biased results ■ Not clinically relevant model	■ Larger representative sample has to be used for applying ML techniques to produce robust, scalable and unbiased results
Lack of multi-center studies	■ Leads to non-generalizable models	■ Registries having multicenter data from heterogeneous set of patients has to be used in the studies
Lack of diverse data streams used	■ Leads to non-generalizable models	■ Studies with diverse data streams are required that could potentially help in providing personalized healthcare solutions

## Supplementary Materials

The following are available online at https://www.mdpi.com/1424-8220/20/21/6100/s1, Table S1: Machine Learning Terms; Table S2: Distribution of studies for each broad ML category; Figure S1: Classification of ML Techniques used in Hematopoietic Stem Cell Transplantation (HSCT) reviewed studies; Figure S2: Distribution of studies by ML broad categories.

## Abbreviations

ML	Machine Learning
AI	Artificial intelligence
GVHD	Graft-versus-host-disease
ATS	Adaptive treatment strategies
HSCT	Hematopoietic stem cell transplantation
HLA	human leukocyte antigen
SVM	Support vector Machines
EHR	Electronic health record
mHealth	Mobile health
PRISMA	Preferred Reporting Items for Systematic Reviews and Meta-Analyses.
LR	Logistic Regression
RF	Random Forest
PCA	Principal Component Analysis
BLSTM	Bidirectional Long-short-term-memory
ADT	Alternating Decision Tree
NB	Naïve Bayes
MLP	Multiplayer perceptron
RL	Reinforcement Learning
CART	Classification and Regression Trees
BRT	Boosted Regression Trees
SR	Spline regression
BART	Bayesian additive regression trees
NN	Neural networks
k-NN	k-nearest neighbor
LDA	Linear Discriminant analysis
SDA	Shrinkage Discriminant analysis
RSF	Random survival Forest
BN	Bayesian Network
GBM	Gradient Boosting Machines
DT	Decision Tree
BL	Bayesian Learners
EL	Ensemble learners
AML	Acute Myeloid Leukemia
CBC	Complete blood count
WBC	White blood clount
rDRI	refined disease risk index
MM	multiple myeloma
NRM	Non-relapse mortality
SOM	Self-organizing map
GVHD-DE	Graft-versus-host-disease- dry eye
VT	Verification Typing
BDT	Boosted decision Trees
AUC	Area under curve
GPS	Generalized path seeker
API	Application programming Interface
PRO	Patient reported outcome
HRQOL	Health related quality of life
HIPPA	Health Insurance Portability and Accountability Act
