# Supplementary material for: A Systematic Review of Machine Learning Techniques in Hematopoietic Stem Cell Transplantation (HSCT)
Source: Sensors (Basel). 2020 Oct 27;20(21):6100. doi: 10.3390/s20216100 (PMC7663237; doi:10.3390/s20216100)
Supplement: Supplementary file 1 [file sensors-20-06100-s001.pdf]

*sensors*

# Supplementary Materials

# A Systematic Review of Machine Learning Techniques in Hematopoietic Stem Cell Transplantation (HSCT)

**Vibhuti Gupta [1,*], Thomas M. Braun [2], Mosharaf Chowdhury [3], Muneesh Tewari [4,5,6] and Sung Won Choi [1,*]**

[1] Michigan Medicine, Department of Pediatrics, University of Michigan; Ann Arbor, 48109 MI, USA
[2] School of Public Health, Department of Biostatistics, University of Michigan, Ann Arbor, 48109 MI, USA; tombraun@umich.edu
[3] Michigan Engineering, Computer Science and Engineering, University of Michigan, Ann Arbor, 48109 MI, USA; mosharaf@umich.edu
[4] Michigan Medicine, Department of Internal Medicine, Hematology/Oncology Division, University of Michigan, Ann Arbor, 48109 MI, USA; mtewari@med.umich.edu
[5] Center for Computational Medicine and Bioinformatics, University of Michigan, Ann Arbor, 48109 MI, USA
[6] Michigan Engineering, Department of Biomedical Engineering, University of Michigan, Ann Arbor, 48109 MI, USA
* Correspondence: gvibhuti@med.umich.edu (V.G.); sungchoi@med.umich.edu (S.W.C.)

Received: 16 September 2020; Accepted: 25 October 2020; Published: date

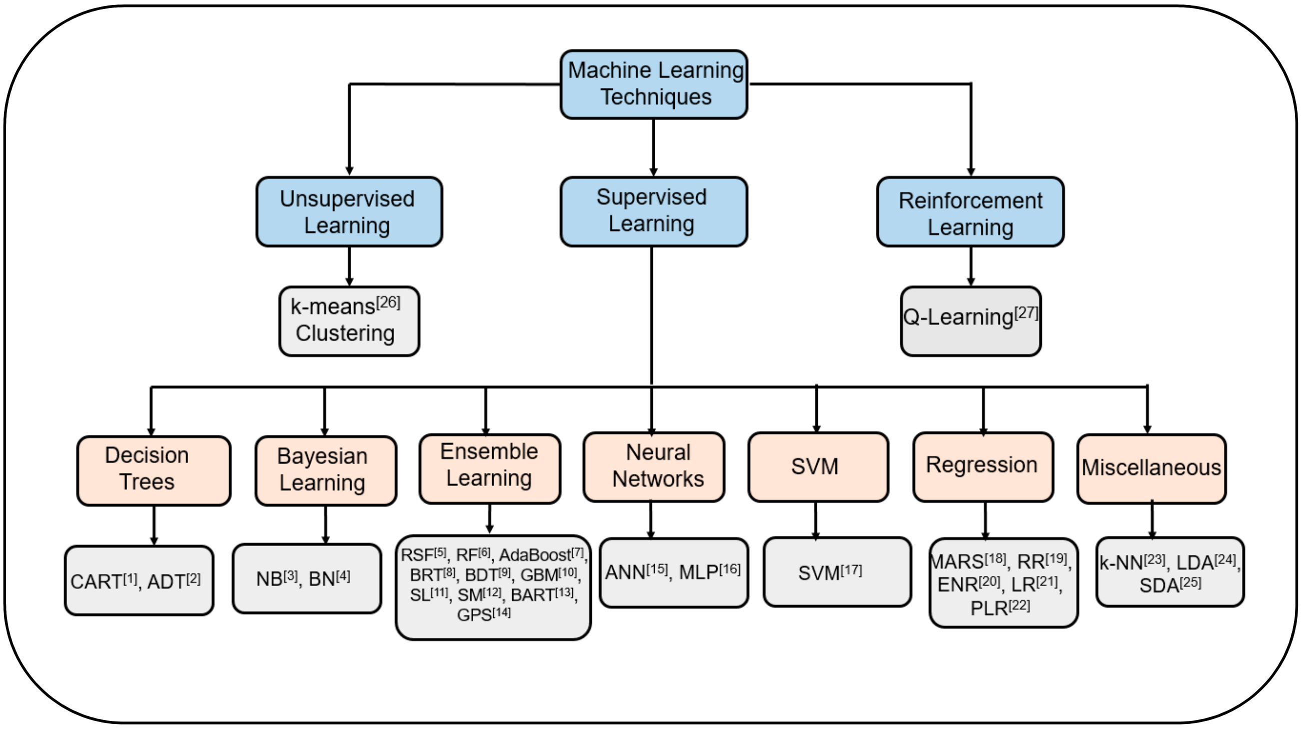


**Figure S1.** Classification of ML Techniques used in Hematopoietic Stem Cell Transplantation (HSCT) reviewed studies.

Note: Superscript numbers in the grey boxes refer to the references below. Deep learning techniques are not shown here since they are the subset of Machine Learning but not coming under the types of Machine Learning techniques.

Abbreviated Terms: CART: Classification and Regression Tree; ADT: Alternating Decision Tree; NB: Naïve Bayes; BN: Bayesian Learning; RSF: Random Survival Forest; RF: Random Forest; AdaBoost: Adaptive Boosting; BRT: Boosted Regression Trees; BDT: Boosted Decision Tree; GBM: Gradient Boosting Machine; SL: Super Learner; SM: Stacked Model; BART: Bayesian Additive Regression Tree; GPS: Generalized Path Seeker; ANN: Artificial Neural Network; MLP: Multilayer Perceptron; SVM: Support Vector Machine; MARS: Multivariate Adaptive Regression Spline; RR: Ridge Regression; ENR: Elastic Net Regression; LR: Logistic Regression; PLR: Penalized Logistic Regression; k-NN: k-nearest Neighbor; LDA: Linear Discriminant Analysis; SDA: Shrinkage Discriminant Analysis

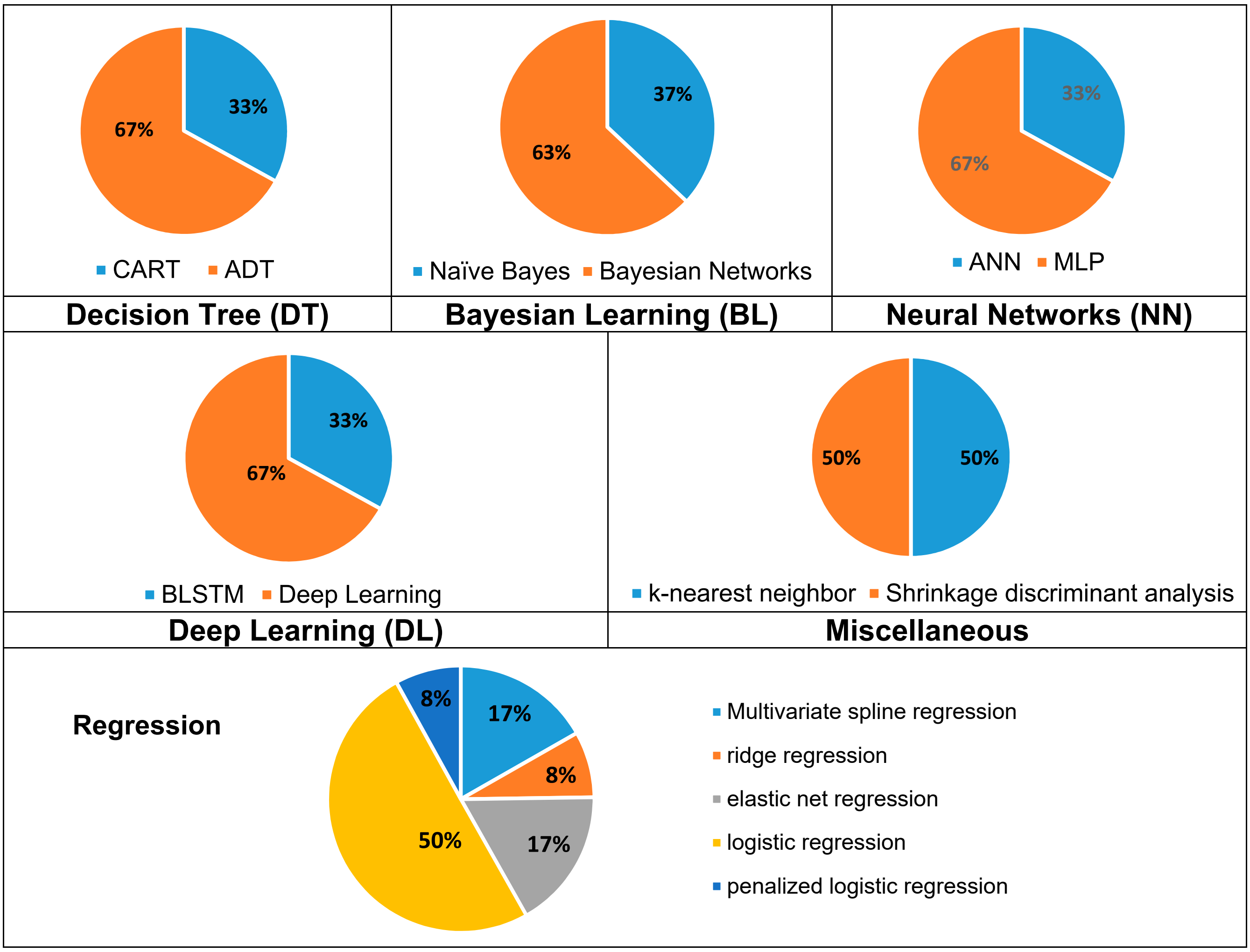

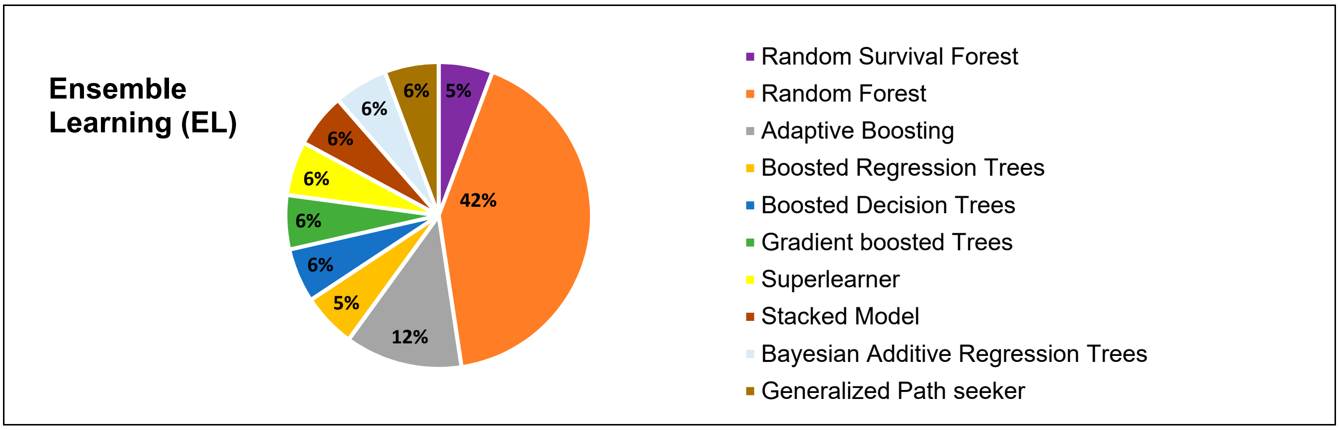


**Figure S2.** Distribution of studies by ML broad categories.

Figure S2 shows the percentage of ML Techniques coming under each broad ML category. Each of the percentage reported are the percentage of ML Techniques in their respective Broad ML Category. For example: ADT ML Technique is used in 67% of studies coming under category Decision Tree (Broad ML category). The most frequently used ML techniques were Alternating decision tree in decision trees (67%), Bayesian networks in BL (63%), random forest and adaptive boosting in EL (42% and 12% respectively), multilayer perceptron in neural networks (67%), k-means clustering (67%) and LR in Regression category (50%). Abbreviated Terms: CART: Classification and Regression Tree, ADT: Alternating Decision Tree, ANN: Artificial Neural Network, MLP: Multilayer Perceptron, BLSTM: Bidirectional Long-short-term-memory, RF: Random Forest, LR: Logistic Regression, BN: Bayesian Networks

**Table S1.** Machine Learning Terms.

| Terms | Description |
|---|---|
| Supervised Machine Learning | A set of ML techniques that requires labels to map input variables into output. |
| Unsupervised Machine Learning | A set of ML techniques identifying patterns in data without known labels |
| Deep Learning | A set of ML techniques based on artificial neural networks that uses multiple layers to extract higher level features from input data |
| Classification and Regression Tree (CART) [1] | A decision tree learning technique that produces a classification or regression tree based on the type of dependent variable (i.e., categorical or numerical) |
| Alternating Decision Trees (ADT) [2] | A generalized version of decision trees that uses boosting and generate smaller and interpretable rules. |
| Naïve Bayes (NB) [3] | A supervised ML technique based on Bayes theorem considering independence assumption between the features |
| Bayesian Network (BN) [4] | Probabilistic graphical models using Bayesian inference |
| Random Survival Forest (RSF) [5] | An ensemble learning technique applicable to survival data and is an extension of random forest. |
| Random Forest (RF) [6] | An ensemble learning ML technique that fits multiple trees on random samples of input data and predicts the class based on the combined predictions |
| Adaptive boosting (Ada-boost) [7] | A supervised ML technique based on boosting that convert a set of weak classifiers to strong for classification |

| | |
|---|---|
| Boosted Regression Trees (BRT) [8]/ Boosted Decision Trees (BDT) [9] | An ensemble learning technique that combines regression trees with boosting by building and combining multiple fits to improve performance. |
| Gradient Boosting Machine (GBM) [10] | An ensemble learning technique that uses boosting to convert weak learners to strong using gradients. |
| Super Learner (SL) [11] | An ensemble learning technique that combines the predictions of multiple ML techniques using cross validation and then produce a weighted average of those model predictions. |
| Stacked Learning [12] | An ensemble technique to build first level of predictions from a base ML technique and then use those predictions to predict the outcome. |
| Bayesian Additive Regression Tree (BART) [13] | An ensemble learning technique that sums the contribution of weak learners. |
| Ensemble Learning[14] | A set of ML techniques where multiple models are combined to predict a given outcome. |
| Artificial Neural Network (ANN) [15] | A supervised ML technique that consists of three layers i.e., input, hidden and output layers for processing and mimics human brain structure. |
| Multilayer perceptron (MLP) [16] | A supervised ML technique based on feedforward neural networks |
| Support vector machines (SVM) [17] | A supervised ML technique that transforms the input finite-dimensional space into higher dimensional (hyperplane) by linear or non-linear transformations and can be used for classification or regression |
| Multivariate Adaptive Regression Spline (MARS) [18] | A flexible adaptive regression technique that captures non-linearity in the data automatically and applicable to high dimensional data. |
| Ridge Regression (RR) [19] | A regression technique that is used for multivariate regression in data having multicollinearity among variables |
| Elastic Net Regression (ENR) [20] | A regularized regression method that combines LASSO and Ridge penalties. |
| Logistic Regression [21] | A type of regression which determines the probability/odds of outcome based on the combination of predictors |
| k-nearest neighbor (k-NN) [23] | A supervised ML Technique that labels the given input data point based on the labels of the nearest neighbors defined by k. |
| Linear discriminant Analysis (LDA) [24] | A dimensionality reduction technique that determines a linear combination of features to maximize the class separation |
| Shrinkage Discriminant Analysis (SDA) [25] | A dimensionality reduction technique that adds a shrinkage parameter to linear discriminant analysis for its applicability in high dimensions. |
| k-means [26] | An unsupervised ML technique that clusters the given data set by using the distance metrics. |
| Reinforcement Learning [27] | Type of ML technique that produces a sequence of decisions and continually learns based on the prior decisions to maximize the reward. |
| Decision Trees (DT) [28] | A supervised ML technique that extracts a set of rules to predict the labels for a given input data |
| Bagging | An ensemble technique that combines the predictions of multiple models to predict a new input data instance |
| Boosting | An ensemble technique based on boosting the weak learners to improve predictions |

Note: Numbers in brackets are referred to below references

**Table S2.** Distribution of studies for each broad ML Category.

| ML Category | No. of studies[1] | Percentage[2] |
|---|---|---|
| Decision Tree | 6 | 22 |
| Bayesian Learning | 8 | 30 |
| Ensemble Learning | 17 | 63 |
| Neural Networks | 3 | 11 |
| Support Vector Machine | 8 | 30 |
| Clustering | 3 | 11 |
| Deep Learning | 3 | 11 |
| Regression | 12 | 44 |
| Miscellaneous | 2 | 7 |
| Reinforcement Learning | 2 | 7 |

[1]Total number of studies are not equal to total number of reviewed studies (27) here since multiple ML Techniques were used in each study.
[2]Percentage is calculated as the number of studies coming under each broad category divided by the total number of studies (27).

## References


1. Loh, W. The alternating decision tree learning algorithm. **2011**, *1*, 14–23, doi:10.1002/widm.8.
2. Freund, Y.; Mason, L. The alternating decision tree learning algorithm. *Proceedings of the Sixteenth International Conference on Machine Learning*, Morgan Kaufmann: San Francisco, USA, 1999, pp. 124-133.
3. Mitchell T. *Machine Learning*. McGraw-Hill: New York, USA, 1997.
4. Heckerman, D. A tutorial on learning with Bayesian networks. *Innovations in Bayesian networks*, Springer: Berlin, Heidelberg, 2008, pp. 33-82.
5. Ishwaran, H.; Kogalur, U.B.; Blackstone, E.H.; Lauer, M.S. Random survival forests. *Ann. Appl. Stat.* **2008**, *2*, 841–860, doi:10.1214/08-aoas169.
6. Breiman, L. Random forests. *Mach. Learn.* **2001**, *45*, 5–32.
7. Rojas, R. AdaBoost and the super bowl of classifiers a tutorial introduction to adaptive boosting. Available online: http://www.inf.fu-berlin.de/inst/ag-ki/rojas_home/documents/tutorials/adaboost4.pdf (Accessed on 10 September 2020)
8. Elith, J.; Leathwick, J.R.; Hastie, T. A working guide to boosted regression trees. *J. Anim. Ecol.* **2008**, *77*, 802–813, doi:10.1111/j.1365-2656.2008.01390.x.
9. Drucker, H.; Cortes, C. Boosting decision trees. In Proceedings of the In Advances in neural information processing systems, Denver, CO, USA, 2-5 December 1996; pp. 479-485.
10. Natekin, A.; Knoll, A. Gradient boosting machines, a tutorial. *Front. Neurorobotics* **2013**, *7*, 21, doi:10.3389/fnbot.2013.00021.
11. Van Der Laan, M.; Polley, E.; Hubbard, A. Super Learner. *Stat. Appl. Genet. Mol. Biol.* **2007**, *6*, doi:10.2202/1544-6115.1309.
12. Wolpert, D.H. Stacked generalization. *Neural Networks* **1992**, *5*, 241–259, doi:10.1016/s0893-6080(05)80023-1.
13. Chipman, H.A.; George, E.I.; McCulloch, R.E. BART: Bayesian additive regression trees. *Ann. Appl. Stat.* **2010**, *4*, 266–298, doi:10.1214/09-aoas285.
14. Friedman, J.H. Fast sparse regression and classification. *Int. J. Forecast.* **2012**, *28*, 722–738, doi:10.1016/j.ijforecast.2012.05.001.
15. Krogh, A. What are artificial neural networks? *Nat. Biotechnol.* **2008**, *26*, 195–197, doi:10.1038/nbt1386.
16. Murtagh, F. Multilayer perceptrons for classification and regression. *Neurocomputing* **1991**, *2*, 183–197, doi:10.1016/0925-2312(91)90023-5.
17. Cortes, C.; Vapnik, V. Support-vector networks. *Mach. Learn.* **1995**, *20*, 273–297, doi:10.1007/bf00994018.
18. Friedman, J.H. Multivariate Adaptive Regression Splines. *Ann. Stat.* **1991**, *19*, 1–67, doi:10.1214/aos/1176347963.

19. Jain, R. Ridge regression and its application to medical data. *Comput. Biomed. Res.* **1985**, *18*, 363–368, doi:10.1016/0010-4809(85)90014-x.
20. Zou, H.; Hastie, T. Regularization and variable selection via the elastic net. *J. R. Stat. Soc. Ser. B Statistical Methodol.* **2005**, *67*, 301–320, doi:10.1111/j.1467-9868.2005.00503.x.
21. Peng, C.-Y.J.; Lee, K.L.; Ingersoll, G.M. An Introduction to Logistic Regression Analysis and Reporting. *J. Educ. Res.* **2002**, *96*, 3–14, doi:10.1080/00220670209598786.
22. Tibshirani, R. Regression Shrinkage and Selection Via the Lasso. *J. R. Stat. Soc. Ser. B (Statistical Methodol.* **1996**, *58*, 267–288, doi:10.1111/j.2517-6161.1996.tb02080.x.
23. Zhang, Z. Introduction to machine learning: k-nearest neighbors. *Ann. Transl. Med.* **2016**, *4*, 218, doi:10.21037/atm.2016.03.37.
24. McLachlan, G.J. *Discriminant analysis and statistical pattern recognition*, John Wiley and Sons: Hoboken, NJ, USA,2004.
25. Hastie T; Tibshirani, R; Friedman J. *The Elements of Statistical Learning: Data Mining, Inference, and Prediction*. Springer Science and Business Media; Berlin, Germany, 2009.
26. Jin X.; Han J. *K*-Means Clustering. In: Sammut C., Webb G.I. (eds) *Encyclopedia of Machine Learning*. Springer: Berlin, Germany, 2011.
27. Kaelbling, L.P.; Littman, M.L.; Moore, A.W. Reinforcement Learning: A Survey. *J. Artif. Intell. Res.* **1996**, *4*, 237–285, doi:10.1613/jair.301.
28. Quinlan, J. Simplifying decision trees. *Int. J. Man-Machine Stud.* **1987**, *27*, 221–234, doi:10.1016/s0020-7373(87)80053-6.
